# Differentiating Human Falls from Daily Activities Using Machine Learning Methods Based on Accelerometer and Altimeter Sensor Fusion Feature Engineering

**DOI:** 10.3390/s25237220

**Published:** 2025-11-26

**Authors:** Krunoslav Jurčić, Ratko Magjarević

**Affiliations:** Faculty of Electrical Engineering and Computing, University of Zagreb, 10000 Zagreb, Croatia; ratko.magjarevic@fer.hr

**Keywords:** fall detection, human activity recognition, sensor fusion, machine learning, signal processing, accelerometer, barometric altimeter

## Abstract

This paper presents a detailed analysis of signal data acquired from wearable sensors such as accelerometers and barometric altimeters for human activity recognition, with an emphasis on fall detection. This research addressed two types of activity recognition tasks: a binary classification problem between activities of daily living (ADLs) and simulated fall activities and a multiclass classification problem involving five different activities (running, walking, sitting down, jumping, and falling). By combining features derived from both sensors, traditional machine models such as random forest, support vector machine, XGBoost, logistic regression, and majority voter models were used for both classification problems. All of the aforementioned methods generally produced better results using combined features of both sensors compared to single-sensor models, highlighting the potential of sensor fusion approaches for fall detection.

## 1. Introduction

In recent times, human activity recognition (HAR) has become a research area that attracted a great deal of interest, especially within the fields of medicine, healthcare, and biomedical engineering. One of the key factors contributing to this growth is the increasing availability of data collected from many devices, such as smartphones and mobile apps, portable sensors, wristbands, and smart watches, which facilitate continuous monitoring and real-time analysis. Statistical analyses of biological data acquired from various sensors can provide useful information for a wide range of medical applications. In a rapidly aging society, elderly care and rehabilitation represent a significant challenge for public healthcare systems. According to data from the World Health Organization (WHO), falls rank as the second most common cause of unintentional injury-related fatalities on a global scale [1]. Moreover, it is also important to stress the fact that individuals aged 60 and above experience the highest incidence of fatal falls. This particularly poses a threat for older adults living independently, as living on one’s own can place elderly people at increased risk when it comes to performing daily activities due to a decline in motor skills. Apart from physical damage, it could leave psychological consequences as well, as around 60 percent of older people are affected psychologically by falls [2]. Therefore, it is essential to develop reliable and effective fall detection systems with the goal of reducing the considerable medical and economic consequences of falls while simultaneously helping healthcare providers and promoting broader well-being [3].

Fall detection as a research area has attracted interest in recent years since users generate real-time data from portable devices that may be wearable (attached to the subject’s body) or non-wearable (e.g., cameras, pressure sensors, ultrasound, or optical motion sensors) [4]. Methods based on wearable sensors offer advantages in terms of cost, size, weight, power consumption, ease of use, and portability. The applied fall detection methods could be classified into three main categories: (1) threshold; (2) traditional machine learning; and (3) deep learning methods [5]. Many studies have researched the integration of HAR algorithms by analyzing publicly available wearable-sensor datasets captured in real-world settings [6,7,8,9,10]. Other researchers have focused on designing new devices and datasets to further broaden our understanding of human activity recognition [11,12]. The integration of fall detection systems into Internet of Things (IoT) environments also attracted significant interest. By leveraging interconnected devices and cloud-based infrastructure, IoT-enabled solutions facilitate continuous monitoring, real-time data transmission, and remote accessibility to caregivers and healthcare professionals. Building on these capabilities, fall detection devices can seamlessly interact with other smart technologies within domestic or clinical environments, thereby establishing a comprehensive safety network around individuals. Such integration not only enables automated alerts to be transmitted to family members, medical professionals, or emergency services but also allows coordination with IoT-enabled systems—such as lighting and door access controls—to support quick assistance and improve overall response efficiency [13,14,15,16,17].

Regarding the type of biomedical signal data typically used, accelerometers remain the most widely utilized for the development and deployment of fall detection algorithms because of their low cost, energy efficiency, and ability to capture motion dynamics with high temporal resolution. However, numerous studies have also investigated the integration of alternative sensors such as gyroscopes, magnetometers, barometers, and physiological sensors to complement accelerometer data. Sensor fusion approaches have also been brought to attention, using combined information from multiple heterogeneous sensors to improve the robustness, accuracy, and reliability of fall detection systems. Sensor fusion models could prove to be particularly valuable in reducing false alarms and enhancing generalization [18]. Although not nearly as used as accelerometers and with open-access datasets being scarce, barometric altimeter analysis is thought to be a useful complement to accelerometers for building fall detection systems, with their potential of detecting changes in elevation [19,20]. Wang et al. proposed a fall detector based on accelerometers and barometers [21]. Despite its quite low power consumption (about two years of autonomy), the specificity of this fall detector was 87.3% when evaluated on unseen test sets. Sabatini et al. used three sensors in their fall detector. They designed an extended Kalman filter to fuse the measurements of accelerometers and gyroscopes in order to estimate the orientation of the wearable device [22]. To our knowledge, no significant research or publicly available datasets regarding the collection and analysis of both accelerometers and barometric altimeters for human activity recognition problems have been published since the FallAllD dataset by Saleh et al. in 2021 [11].

The authors of this paper have been exploring the usage of traditional machine learning methods for fall detection. Some of our previous research in this field included performing both supervised and unsupervised methods, comparing the performances of clustering and prediction on features derived from signals acquired from wearable sensors while also taking into account on the interpretability of the deployed models [23]. We conducted multiclass classification using a dataset (“UniZg activ2”) consisting of accelerometer, magnetometer, and gyroscope recordings of twelve activities, nine of which describe activities of daily living (“standing”, “sitting down”, “walking”, “standing up”, “walking downstairs”, “walking upstairs”, “lying down”, “running”, and “jumping”) and three describing activities of simulated falls (“falling forward”, “falling backward”, and “side falling”), with a focus being on fall detection [24]. The analysis showed that accelerometer-derived features performed significantly better than gyroscope and magnetometer features, with the latter producing the poorest results. The analysis also showed that most of the falsely classified falls were mistaken for sitting down, probably due to similar body movement described by the accelerometer signal. For the next data collecting session, the authors decided to focus on the potential of barometric altimeters, believing that they can help improve the differentiation of falling from other activities of daily living. Using accelerometer data as a reference, we conducted an approximate detection of the impact phase using altitude averages of barometric altimeter signals [25]. The research results showed that these features successfully approximated the impact phase and that altimeter data can prove to be useful in fall detection problems. The authors have collected data from various activities acquired using wearable sensors, with an aim to expand the global database of multisensor biomedical signal data for human activity detection. The presented study describes our research on the sensor fusion of the aforementioned sensors (accelerometers and barometric altimeters) for the recognition of fall activities and their differentiation from activities of daily living in order to evaluate whether barometric altimeter integration leads to better and more interpretable results.

## 2. Materials and Methods

**Figure 1:**
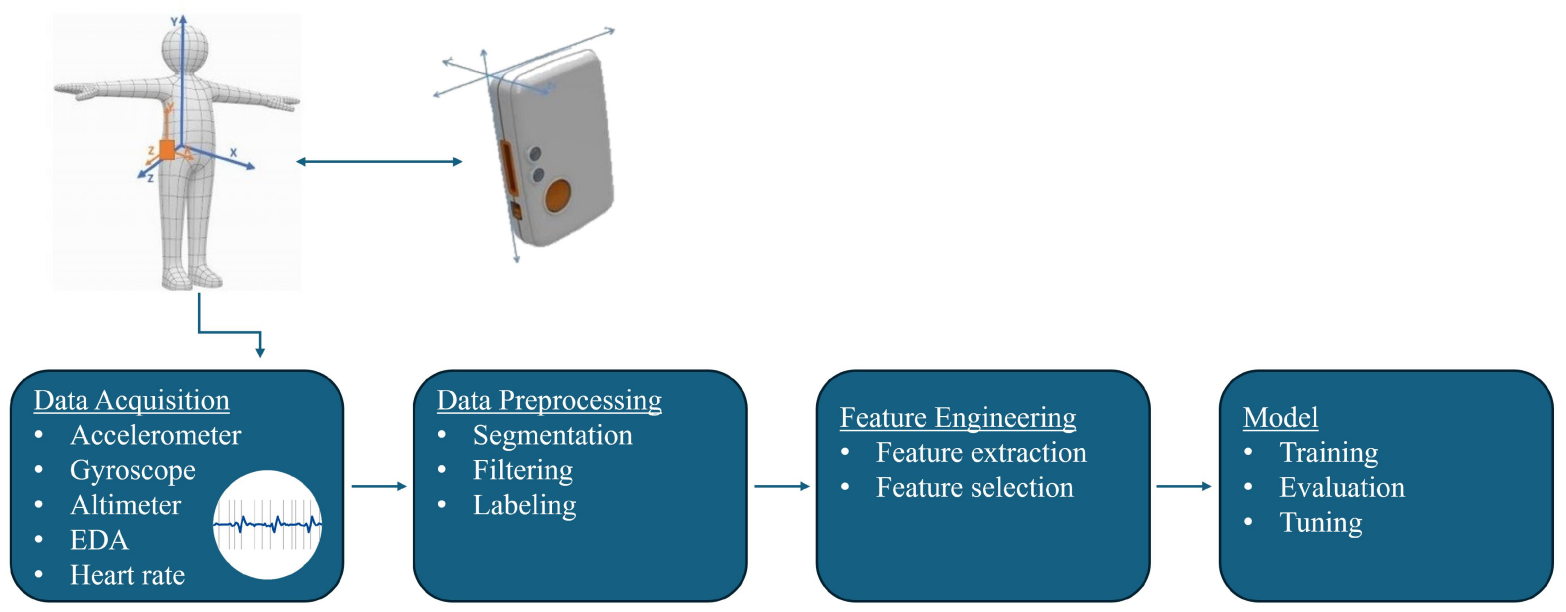
Proposed fall detection model pipeline.

This research consisted of several stages, including data acquisition, data preprocessing, feature engineering, and model training and evaluation. The proposed fall detection model pipeline is presented in Figure 1.

### 2.1. Datasets

The dataset used in this research is a combination of three separate datasets, all acquired by the authors on the premises of the University of Zagreb, Faculty of Electrical Engineering and Computing, on separate occasions, collecting signal data of both activities of daily living (ADLs) and simulated falls. The activities were recorded using Shimmer3 (Shimmer Sensing, Dublin, Ireland) devices, which are battery-powered wireless sensor nodes equipped with multiple sensors [26]. Each sensor node consists of multiple MEMS sensors, which include two 3D accelerometers (a wide-range accelerometer and a low-noise accelerometer), a 3D magnetometer, a 3D gyroscope, and devices for measuring barometric pressure, temperature, electrodermal activity (EDA), and heart rate (HR), as well as a Bluetooth module for real-time data streaming. Two different types of sensor nodes were used for data acquisition: Shimmer3 Inertial Measurement Unit (IMU) and Shimmer3 Galvanic Skin Response (GSR+) unit. For all conducted experiments, the authors used a wide-range accelerometer with a measurement range set to ±8 g, a magnetometer, and a gyroscope, as mentioned in the earlier research by Šeketa et al. [27], with the addition of a barometric altimeter. However, the magnetometer and gyroscope recordings, as well as EDA and HR signal data recordings, were excluded from the present analysis. All sensors were sampled at a frequency $f_{s}$ of 201 Hz. An example of sensor placement regarding both sensor node units is shown in Figure 1. Although they were given oral instructions, it was important for the subjects to place the sensors themselves to make the recordings more trustworthy, because, in real-life applications, users of wearable fall detection systems should also be able to place the sensors without the assistance of a professional. All participants gave informed consent before participating in this study, and the study protocol was approved by the Ethics Committee at the University of Zagreb, Faculty of Electrical Engineering and Computing.

#### 2.1.1. UniZgFall1 Dataset

The first dataset included in this study, the UniZgFall1 dataset, consists of solely simulated fall recordings of 39 healthy subjects who were recruited to participate voluntarily, 12 of whom were female and 27 male. All participants gave informed consent before participating in this study, and the study protocol was approved by the Ethics Committee at the University of Zagreb, Faculty of Electrical Engineering and Computing. The mean and standard deviation for the age, height, and weight of the subjects were 27.9 ± 2.7 years. The subjects wore three Shimmer3 IMU devices [26]. Two of them were placed above the navel, and the third was placed on the subjects’ right hip, at the height where they would wear a belt. After the sensor node’s placement, the subjects performed five different simulated fall experiments. The first two fall activities were performed after only verbal instructions (no example videos or demonstrations were provided), and no warm-up trials were allowed in the hope of avoiding “adaptation” of the movements, which then makes the falls seem less natural. Before the third and fourth experiments, subjects were shown video clips of persons falling and were asked to reproduce the falls shown in the videos. Both videos were selected from publicly available sources and included a fall to the back when missing a chair while attempting to sit, similarly to the first and second experiments. The video recording of a simulated fall used for the third experiment is from the publicly available SisFall dataset [28]. The fourth experiment used a video of a real-world fall recorded during the study by Robinovitch et al. as an example [29]. The recording shows an elderly person falling on his back while trying to sit down in a wheelchair. Since the aforementioned person in the recording was holding on to a table while trying to sit down, the subjects were asked to repeat this activity by holding on to an object while lowering to sit down for the fifth and final experiment. The order of execution of the experiments varied for every subject.

#### 2.1.2. SitFall Dataset

The SitFall dataset includes recordings of 253 healthy subjects. The recruiters were mostly students at the University of Zagreb, who agreed to participate voluntarily. All participants gave informed consent before participating in this study, and the study protocol was approved by the Ethics Committee at the University of Zagreb, Faculty of Electrical Engineering and Computing. The subjects include 114 female and 139 male subjects, with a mean age and standard deviation of 21.6 ± 1.4 years. The subjects wore a Shimmer3 IMU sensor node above the navel and performed activities of daily living by sitting down on three chairs with different heights and properties and simulating fall activities [26]. The protocol included two static chairs of different heights and a rotating chair. The simulated fall part of the protocol was similar to a part of the UnizgFall1 dataset protocol: the instruction given to the participants was to fall “as if they were missing sitting on a chair”. The dataset includes recorded signals from the accelerometer, gyroscope, and barometric altimeter.

#### 2.1.3. HAS Dataset

The last dataset, preliminarily named the HAS (Human Activities and States) dataset, was acquired both at the Laboratory of Biomedical Electronics of the University of Zagreb, Faculty of Electronical Engineering and Computing, and at the FootLab Laboratory of Pontifical Javierian University in Bogota, Colombia. The dataset consists of accelerometer, gyroscope, temperature, barometric pressure, electrodermal activity (EDA), and heart rate (HR) signal data, all of which were acquired using the Shimmer3 GSR+ Unit wearable sensor worn on the subject’s wrist [26]. The dataset currently contains data from 26 healthy subjects, with the goal of expanding the dataset to at least 50 subjects and making the data publicly available. The current subject list includes 8 female and 18 male subjects. Their mean age and standard deviation are 29.6 ± 9.8 years. The protocol of data acquisition was inspired by Birjandtalab et al. [30], and it consists of the following:

- Physical Stress: Subjects perform various physical activities of daily living (sitting on two chairs, walking, running, and jumping) and simulated falling.
- Relaxation: One minute.
- Emotional Stress: 5 min. The subject is shown a short horror movie (“Don’t look away”).
- Second Relaxation: One minute.
- Cognitive Stress: The subject performed the Stroop test. The Stroop test consisted of reading the names of colors written in a different colored ink and then saying what color the ink was. After this, the subject performed the Trail Making Test (TMT). It involves connecting a series of circles or squares (representing numbers and/or letters) in a specific order as quickly as possible while maintaining accuracy. The test is divided into two parts: Part A focuses on connecting numbers in order, while Part B requires alternating between numbers and letters.

For the purpose of this study, only the “Physical Stress” part of the dataset was segmented and used.

### 2.2. Data Preprocessing

**Figure 2:**
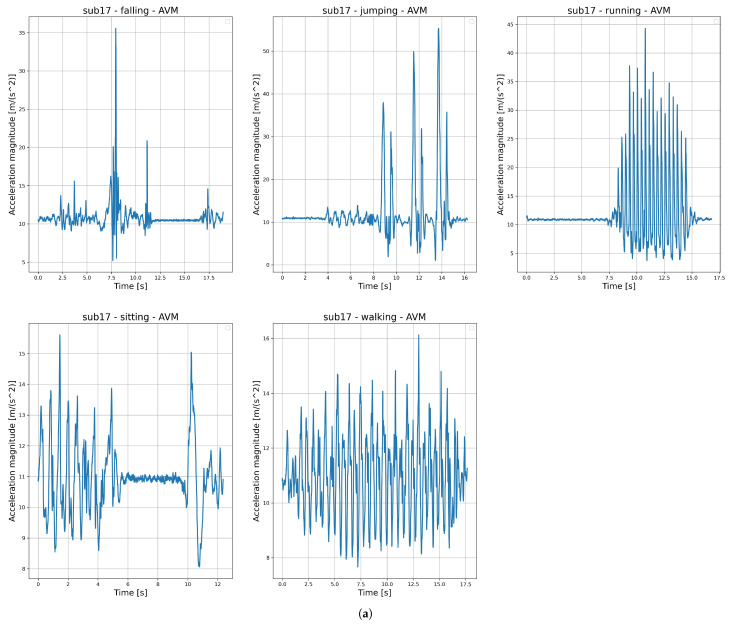

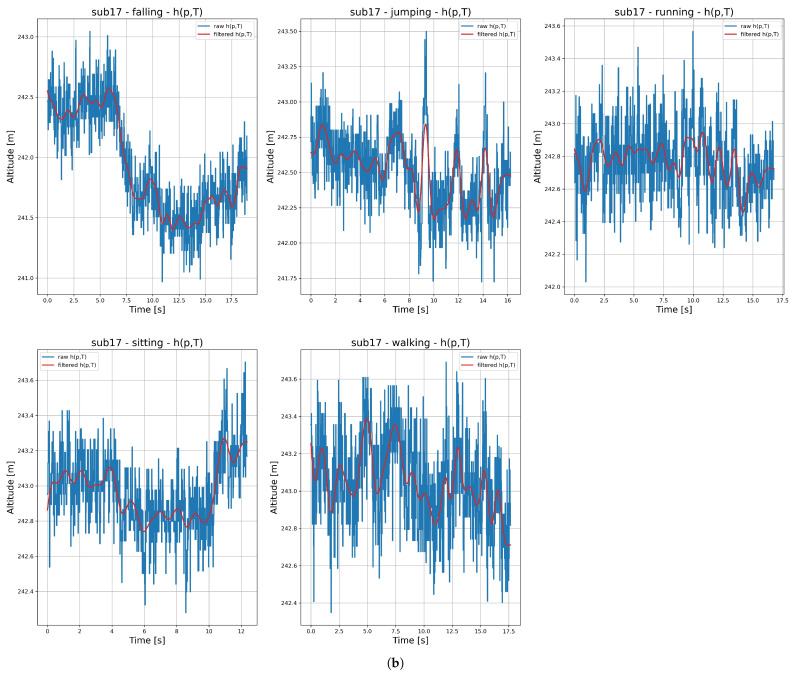
An example of (**a**) acceleration vector magnitude signal AVM and (**b**) barometric altitude signal h(p,T) (raw and filtered) illustrating five different human activities: falling, jumping, running, sitting, and walking.

After acquisition and segmentation of raw data, the next step towards the human fall detection model represents data preprocessing. From the measured raw acceleration data, the acceleration vector magnitude (*AVM*) was calculated to combine the data from all three axes into a single signal. The AVM is obtained using the following equation:

(1) $$AVM\left(n\right)=\sqrt{a_{x}^{2}\left(n\right)+a_{y}^{2}\left(n\right)+a_{z}^{2}\left(n\right)}$$

where *n* represents the current data sample, and $a_{x}\left(n\right)$, $a_{y}\left(n\right)$, and $a_{z}\left(n\right)$ represent its acceleration components along the sensor’s *x*, *y* and *z* axes, respectively. Altitude signal h(p,T) was calculated from the recorded raw barometric pressure and temperature data using the following barometric formula:

(2) $$h\left(p,T\right)=\frac{T+273.15}{0.0065}\left(1-{\left(p/p_{0}\right)}^{0.19}\right)$$

where *p* represents the barometric pressure expressed in kPa; *T* represents the ambient temperature expressed in °C; $p_{0}$ is a constant representing the standard atmosphere pressure at zero level, and it is defined as 101.325 kPa in normal conditions. Since the calculated raw altitude signal is very prone to noise, a Butterworth low-pass filter with a cutoff frequency $\omega_{c}$ of 1 Hz was used. Sensitivity analyses were conducted using several cutoff frequencies (10 Hz, 7 Hz, 5 Hz, 3 Hz, and 1 Hz), and 1 Hz provided the best filtering results. The examples of AVM and h(p,T) signals (raw and filtered) are shown in Figure 2.

### 2.3. Feature Engineering

One of the main focuses of this research included a thoroughly executed feature engineering process to provide an interpretable human activity recognition model, as well as an accurate one. The authors therefore conducted an expanded feature extraction and feature analysis with many features, considering both accelerometer- and altimeter-specific features, as well as more generalized ones. Parts of our previous research also focused exclusively on analyzing time- and frequency-domain features separately for clustering various activities of daily living and simulated falls [23]. However, only accelerometer signal data were processed for the purposes of these clustering problems. This research hypothesizes that the inclusion of altimeter signal features would enhance the model’s ability to differentiate between activities of daily living and falling activities, thus improving classification performance.

#### 2.3.1. Feature Extraction

In total, 55 different features were initially extracted from both accelerometer and altimeter signals, and they can be divided into several categories:

1. Time-domain features:
  - Statistical features (e.g., mean, median, standard deviation, kurtosis, skewness);
  - Peak/trough features (e.g., average peak amplitude, variability of peak amplitudes, average time between steps);
  - Jerk features (e.g., average jerk magnitude, jerk variability, root mean square jerk);
  - Temporal dynamics features (e.g., lag-1 autocorrelation, zero crossing rate);
  - Kinematic features (altimeter-specific) (e.g., mean vertical velocity, max ascent/descent speed, ascent/descent duration).
2. Frequency-domain features
  - Spectral shape (e.g., spectral centroid, spectral spread, spectral entropy).
  - Dominant frequency and power (e.g., dominant frequency, power at dominant frequency).
  - Band power ratios (e.g., high-/low-frequency band power ratio).
3. Energy and complexity features
  - E.g., Shannon entropy, signal energy.

The reason behind selecting various types of features lies in their respective properties used to detect different signal patterns and behaviors.

In physics, jerk (also known as jolt) describes the rate of change with respect to an object’s acceleration over time. Mathematically, it can be expressed as a derivative of acceleration *a* or the third derivative of position *r*:

(3) $$j\left(t\right)=\frac{da(t)}{dt}=\frac{d^{3}r\left(t\right)}{dt^{3}}$$

Since jerk reflects how quickly acceleration changes, jerk features are therefore sensitive to sudden and unexpected movements. For instance, the jerk root mean square measures the overall jerk magnitude (energy) and strongly discriminates abrupt events from steady cyclic activities; therefore, it is a valuable feature for fall detection.

Temporal dynamics features typically focus on the evolution of a signal over time in terms of capturing its periodicity, repetition, or irregularity. Lag autocorrelation quantifies the relationship between a time series and lagged versions of itself, and it is described as follows:

(4) $$R\left(\tau \right)=\sum_{t=1}^{N-\tau }x\left(t\right)\;x\left(t+\tau \right)$$

where τ represents lag or the time difference between the current data point and the past data point. In this case, we used τ = 1, meaning that it compares the current value to the immediately preceding value. Lag autocorrelation detects periodicity with great success and is, as such, a great indicator of rhythm; it is commonly found in the gait cycle. Activities such as running or walking are characterized by strong periodicity (regular autocorrelation), while falls exhibit more irregular dynamics (weak autocorrelation).

**Figure 3:**
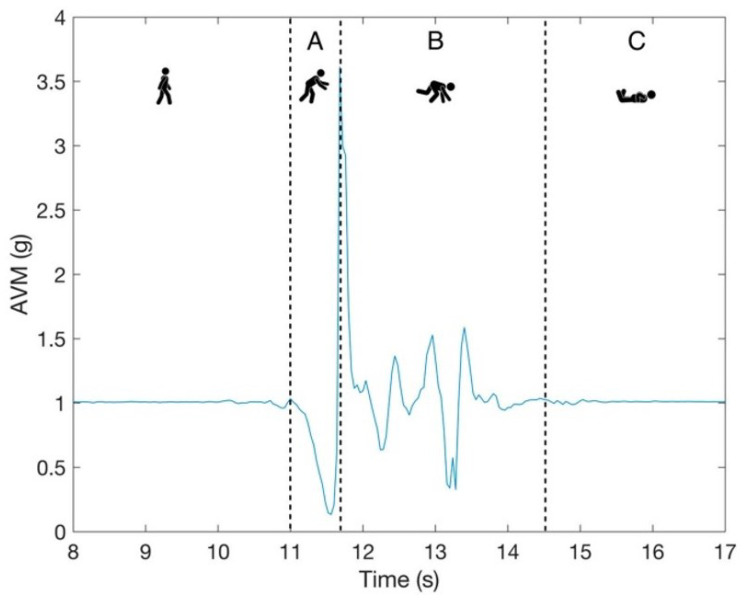
An example of a fall signal with three phases: A is the pre-fall phase, B is the impact phase, and C is the rest phase. The blue line represents expected behaviour of AVM signal during fall phases. The figure was taken from [27].

Kinematic features were derived specifically from altitude h(p,T) signal data since they capture vertical motion. These features help in describing vertical velocity, ascent/descent ratios, and altitude changes, which expand on discrimination between activities that might seem similar for accelerometer data (e.g., walking vs. stair climbing). Since the recorded falls were simulated, the authors cannot help but realize that the expectancy of falling mentally played a role in some subjects who performed them, and thus, the signals could be somewhat less intense compared to real-life falls and more similar to sitting-down activities. In this case, altitude-based features could provide useful information for distinctions. In our earlier research, we examined the approximation of the start of the impact phase of falls, which is marked by part B in the fall model described in Figure 3.

Using only simulated fall signals from the UniZgFall1 dataset, AVM data were used as a reference point, and their peak was considered the start of the impact phase [25]. Barometric altimeter data were used for approximation, with altitude averages of two different time windows: $w_{1}=1.25$ s and $w_{2}=2.5$ s. One of the main features used for approximation analysis was Δh, which represents the maximum difference between two subsequent altitude average values expressed in meters:

(5) $$\Delta h=\max(\bar{h}\left[i\right]-\bar{h}\left[i+1\right])$$

where $\bar{h}\left[i\right]$ represents the current average height value, and $\bar{h}\left[i+1\right]$ represents the subsequent average height value. The feature Δh approximated the AVM peak time, and thus, the fall impact phase’s time was obtained with great accuracy (96.41% for $w_{1}$ and 97.10% for the $w_{2}$ time window). The achieved results encouraged us to include Δh in the list of features for this research.

Apart from time-domain features, we also wanted to investigate the influence of frequency-domain features derived from the signals of both sensors. As already mentioned in the Introduction, some of our previous research in the field of human activity recognition included cluster analyses of accelerometer signal data using frequency-domain features, which we expanded to barometric altimeter sensor data as well. The features were obtained by performing fast Fourier transform (FFT) on the original time series data:

(6) $$X_{k}=\sum_{n=0}^{N-1}x_{n}\;e^{-j\frac{2\pi }{N}kn},\;k=0,1,\ldots ,N-1$$

Frequency-domain analysis can help in describing the periodic or non-periodic nature of motion signals. Alongside the aforementioned widely used statistical features, this study also focused on analyzing the impact of some spectral features, such as spectral centroids, spread, and entropy. Although these features are typically used in audio recognition problems, they have been implemented in accelerometer signal analysis as well [31,32]. An advantage of spectral features, such as spectral entropy, for example, is that unlike features based on raw signal amplitude, it captures the distribution of energy across frequencies, making it less sensitive to sensor placement or activity intensity. Band power ratios were also derived, as they usually differentiate between slow, steady motions from fast and abrupt ones.

#### 2.3.2. Feature Selection

**Figure A1:**
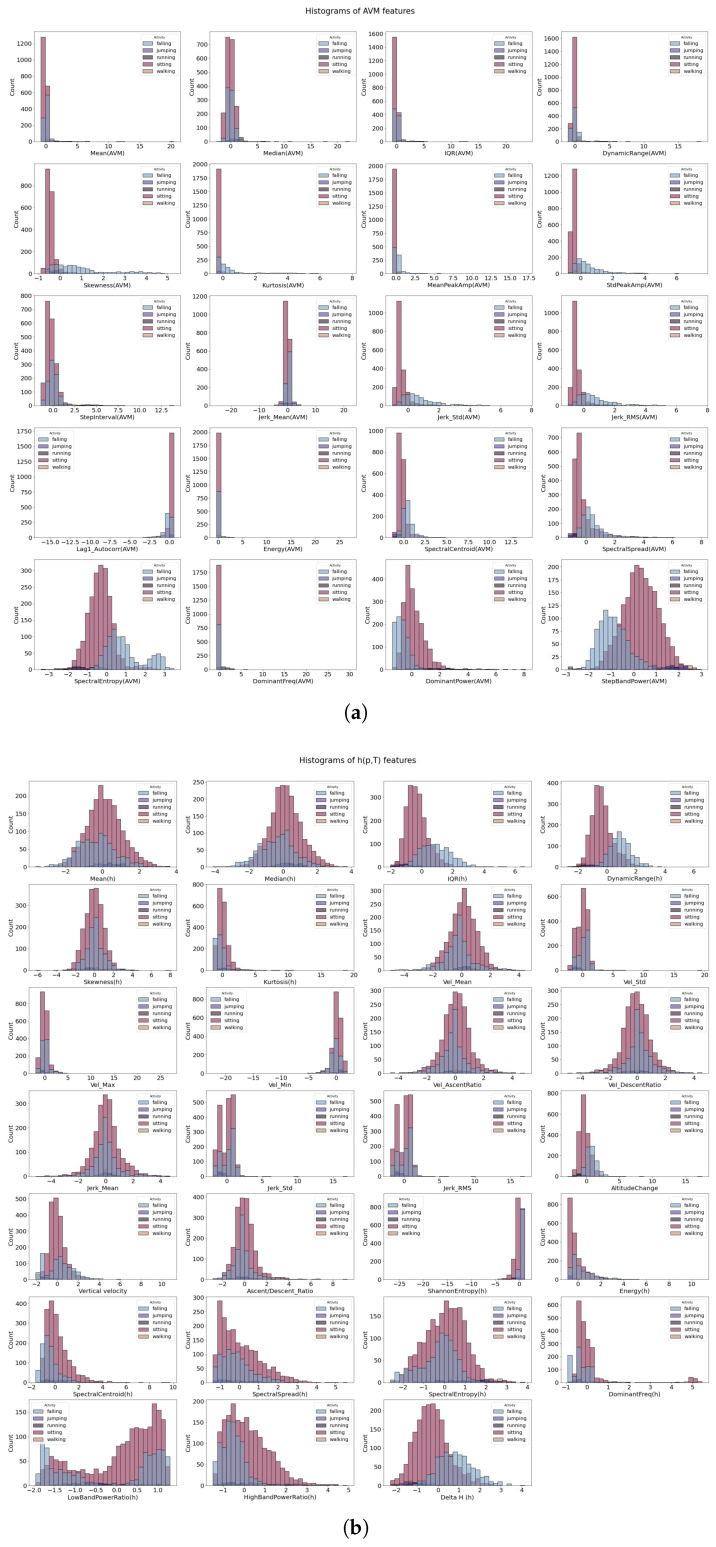
Histograms showing the distribution and variability of extracted features. (**a**) Accelerometer-derived features; (**b**) altitude-derived features.

After the initial features extraction, in order to build simpler and more interpretable models and increase its performance, feature selection was conducted using various data analysis methods. Firstly, the features of each sensor were scaled using *z*-score normalization:

(7) $$z=\frac{x-\mu }{\sigma }$$

where *x* represents the original feature value, μ represents the mean, and σ represents the standard deviation of the feature. Some machine learning models tend to be sensitive to feature magnitude (e.g., logistic regression, SVM, k-NN, distance-based methods, neural networks) and, as such, may assign more importance to features with larger numeric ranges. This type of scaling helps in avoiding this issue, especially when having features derived from two different sensors, as they might display different ranges of values. When using models with interpretable coefficients (such as linear or logistic regression or LASSO), scaling helps in interpretability by ensuring that the coefficients capture the true relative importance of features rather than being dominated by differences in their numerical scales. The histograms of features are shown in Figure A1 in Appendix A, and they help in visualizing the distributions of values among them. The distributions can be helpful in indicating which features could be useful for distinguishing among classes. It is certainly visible that some features show differences in distributions for different classes or, in this case, human activities. The relationships between features were illustrated and analyzed using correlation matrices as well. The matrices were based on Spearman correlation coefficients:

(8) $$\rho =1-\frac{6\sum_{i=1}^{n}d_{i}^{2}}{n\left(n^{2}-1\right)}$$

where $d_{i}$ represents the difference between the two ranks of each observation, and *n* is the number of observations. Spearman correlation was chosen over Pearson correlation since it is based on ranks and not raw values, and it is therefore more robust to outliers and non-normal distributions. The histograms of features show that several features definitely include outliers, so using Spearman’s correlation decreases their influence in the feature selection process. The threshold was set at ρ = 0.85, and through the use of correlation analyses, certain features have proven to be redundant and were therefore removed. The final set of selected features includes 27 features—12 from AVM and 15 from altimeter signals:

- AVM Features: Median; interquartile range (IQR); kurtosis; mean peak amplitude; mean step interval; jerk mean and RMS; signal energy; spectral centroid; spectral spread; spectral entropy; and dominant frequency.
- Altimeter Features: Median; IQR; skewness; kurtosis; mean, maximum, and minimum vertical velocity; jerk RMS; altitude change; vertical velocity; signal energy; spectral centroid; spectral spread; high-frequency band power ratio; and maximum difference between two subsequent altitude average values Δh.

## 3. Results

This study used various traditional machine learning methods, along with an ensemble approach based on majority voting. The models were evaluated on two human activity recognition (HAR) classification tasks. The first task was a binary classification problem distinguishing between activities of daily living (ADLs) and simulated fall activities across all datasets used for training and testing. The second task was a multiclass classification problem, where the ADL category was further divided into distinct activities (sitting, walking, running, jumping, and falling), resulting in a five-class classification problem. In order to make a comparison of results, we performed the classification using sole sensor data and also using sensor fusion of data from both sensors. The traditional machine learning methods used for classifications include the following:

- Random forest (RF);
- Support vector machine (SVM);
- XGBoost (XGB);
- Logistic regression (LR).

In addition, the majority voter (MV) is also used, which makes the classification dependent on the classification of the majority of the aforementioned models. Each of the selected methods has its strengths, making them suitable for good performances regarding the two classification problems, mentioned. The advantage of using classical machine learning methods over deep learning methods such as convolutional and recurrent neural networks, lies in their higher interpretability, providing direct relationships between features and outcomes. The model performance evaluation metrics included 5-fold cross-validation accuracy; weighted and macro-$F_{1}$ scores, alongside precision and recall; and ROC–AUC (receiver operating characteristic–area under the curve):

(9) $$\mathrm{Precision}=\frac{TP}{TP+FP}$$

(10) $$\mathrm{Recall}=\frac{TP}{TP+FN}$$

(11) $$F_{1}=2\cdot \frac{\mathrm{Precision}\cdot \mathrm{Recall}}{\mathrm{Precision}+\mathrm{Recall}}$$

The dataset combining signals from all three datasets used for this analysis includes a total of 3045 recordings of various activities. Among these, sitting on different chairs accounts for 1988 recordings; simulated falls account for 901 recordings; and walking, jumping, and running contribute to 52 recordings each. It is obvious that, especially in multiclass classifications, the issue of imbalances of classes is present. For this reason, the introduction of the macroaveraging of both $F_{1}$ and ROC metrics was deemed necessary by the authors, as it makes the results more trustworthy. Macroaveraging computes the scores of each class independently and then takes their non-weighted mean, thereby giving equal importance to all classes regardless of their sample sizes. While weighted averaging focuses on overall model performance, macroaveraging both metrics ensures that all classes contribute equally, so rare classes are not ignored. Balancing the information received from both would probably be the best approach regarding this particular multiclass problem. Looking at the second classification problem (ADL vs. FALL), the dataset is not as severely imbalanced as in the case of multiple activity classes.

**Figure 4:**
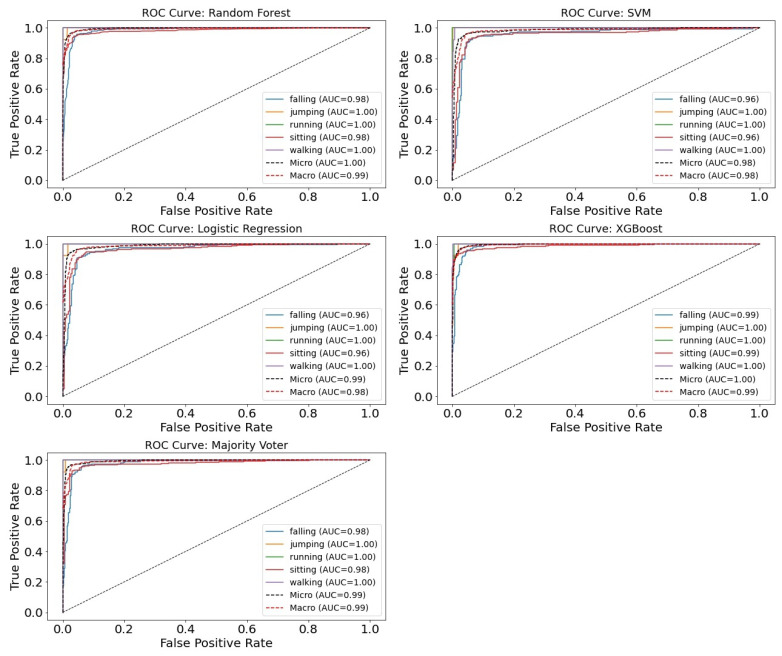
Receiver operating characteristic (ROC) curves for the multiclass classification of human activities across different machine learning models. The curves illustrate class-specific performance (falling, jumping, running, sitting, and walking), together with micro-averaged and macro-averaged ROC curves. The curves illustrate trade-offs between sensitivity (true positive rate) and 1-specificity (false positive rate) across varying decision thresholds. The area under the curve (AUC) is reported as a measure of the model’s overall discriminative ability, with higher AUC values indicating better classification performance.

The visualization of the results included the usage of multiple tables and figures. Table 1, Table 2 and Table 3 show the performance of all machine learning methods when using only AVM features, only altitude h(p,T) features, and a combined feature set derived from the sensor fusion of accelerometers and barometric altimeters using the aforementioned evaluation metrics. The detailed performance of the models regarding class prediction for both classification problems is shown using the ROC curve plots in Figure 4.

**Figure 5:**
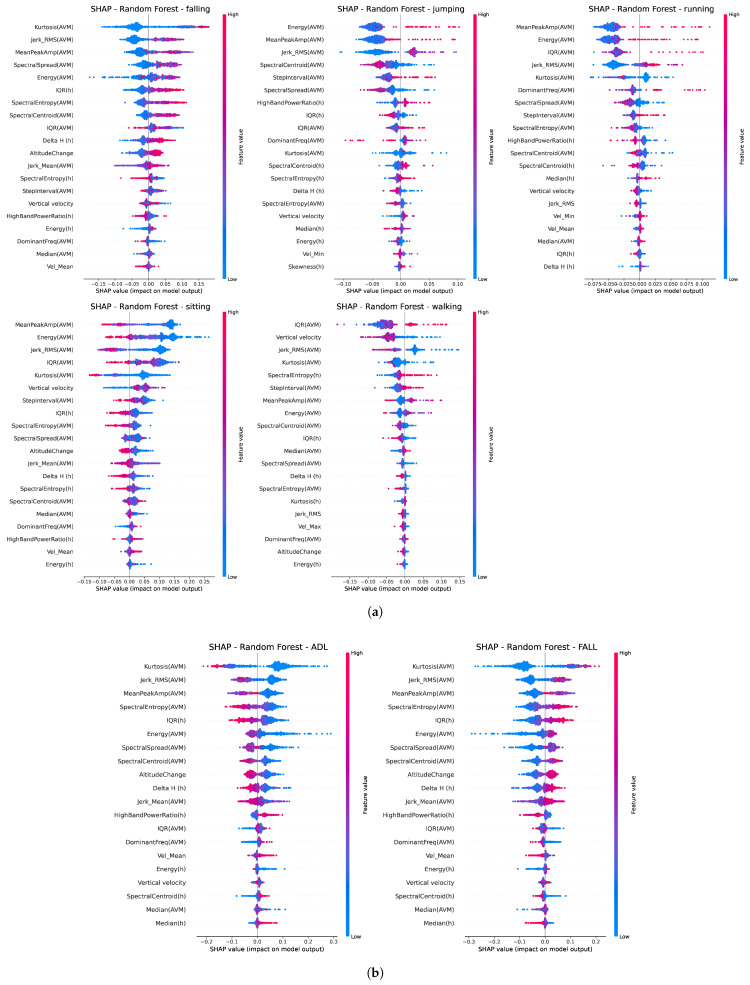
SHAP summary plot illustrating the contribution of individual features to the HAR model using the random forest classification method for (**a**) multiclass classification (falling/jumping/running/sitting/walking) and (**b**) binary classification (ADL/FALL).

In order to improve model interpretability, Shapley Additive Explanations (SHAP) were employed to quantify the contribution of individual features to the model’s predictions, thereby providing insights into the decision-making process of the classifiers. The SHAP visualization of the feature’s contributions across different activities regarding both classification problems using the random forest method is shown in Figure 5. The features are ranked by their overall impact on the model’s output, with colors encoding the magnitude of the corresponding feature value.

## 4. Discussion

The performance of various ML methods used in this study, as seen in Table 1, Table 2 and Table 3, shows that solely using acceleration-derived features provides better classification results than using solely altimeter-derived features. This is mainly due to the altimeter-based model’s poor performance regarding the recognition of jumping, running, and walking activities, affecting its macro-$F_{1}$ results most notably since this metric splits the impact of each class prediction equally. Although these activities were not our main goal of prediction, we must address that they are represented poorly in the dataset, and with less class imbalance, we would expect an improvement in the results regarding their classification. Altimeter-based models, however, seem to be performing well with regard to discriminating between activities of daily living and falling activities and regarding the distinction between falling and sitting activities. The barometric altimeter’s ability to capture the verticality of movement was the primary motivation for its inclusion in fall detection models, since distinguishing between sitting and falling has proven to be one of the most challenging activity-related tasks for accelerometer-only approaches. Notably, combining accelerometer and barometric altimeter data into sensor fusion-based models led to a slight improvement in performance compared to accelerometer-only approaches, thereby enhancing the overall precision of fall detection models. Another important fact to stress lies in high recall results regarding fall prediction (92% to 97% for sensor fusion models). Recall, or sensitivity, is an important metric regarding this particular problem, since it measures how many actual falls are correctly detected. For fall detection, false negatives (missed falls) are the most critical error, and therefore, high recall ensures that the majority of real falls are detected. Among the machine learning methods used in this research, random forest (RF) and XGBoost (XGB) generally achieved slightly superior performance, although all models demonstrated strong overall results, achieving higher overall performance compared to the fall detection model presented by Wang et al. [21].

A possible issue that should be addressed regarding the trustworthiness of models used in this research is presented by the fact that the sensors’ placements while recording the three datasets differ. The signal data used for the recognition of human activities could definitely be affected by sensor placement, as shown in multiple studies [33,34]. Although the subjects recorded during the acquisition of the UniZgFall1 and SitFall datasets shared the same placement position (above the navel), the subjects of the HAS dataset wore the sensors on their wrists, which brings into question whether the differentiation in the sensor placement influenced the overall model performance. We, therefore, conducted cross-dataset generalizations for the SitFall and HAS datasets, since the UniZgFall1 dataset exclusively consisted of fall activities. The generalization was conducted only regarding the binary classification (ADL vs. FALL), as all activity class labels used for the multiclass problem are present only in the HAS dataset, whereas the SitFall dataset only consists of sitting and falling activities. Table 4 shows the summary of cross-data validation results for both of the aforementioned datasets. The results show noticeable dropoffs in performance for the SVM and LR models, while the RF and XGB methods continue to provide strong results, thus showing their robustness. It is interesting to note that the general cross-data generalization of barometric altimeter data shows stronger results than the accelerometer’s data, indicating that the barometric altimeter sensor is less sensitive to its placement when distinguishing between falls and activities of daily living.

Analysis of variance (ANOVA) and Tukey’s HSD (honest significant difference) post hoc tests were performed to determine whether the performance differences among the used machine learning models were statistically significant and, if so, for which ones. The ANOVA test results shown in Table 5 indicated that statistically significant results (p≤0.05) among the models were present regarding the binary classification (ADL vs. FALL) for both accelerometer-based and sensor fusion models, excluding the majority voter (MV). Models trained using altimeter-only features showed no statistically significant differences across classifiers for either classification task, consistent with the results reported in Table 4.

In relation to the influence of features on classification, the SHAP summary plots in Figure 5 show a strong impact of AVM features, such as kurtosis or jerk RMS. This is expected since high kurtosis segments could indicate sudden, sharp changes or bursts, for example, like rapid accelerometer spikes caused by fall impact. High jerk values indicate abrupt movements as well, while low jerk values correspond to smooth and controlled motion. It is noteworthy that the SHAP summary indicated that altimeter features, such as the interquartile range (IQR) and Δh, were highly influential in capturing movement irregularity and the magnitude of vertical movement, thereby contributing to overall model performance.

Regarding future work, there are multiple possible directions for expansion and improvement: One of them includes analyses of wavelet features derived from the signal data. Another aspect of this research that we plan to explore is the evaluation of the model’s robustness regarding noise. The analysis of any data acquired in real time over longer periods of time, including data from wearable sensors, is accompanied by real-life problems, such as noisy or missing values. Our proposed approach to this problem includes adding synthetically created noise to the data and increasing the level of noise gradually to assess the model’s performance. The goal would be to identify the threshold at which noise produces a statistically significant decline in accuracy. Additionally, one of the next steps of our research is also the deployment of deep learning (DL)-based models, such as long short-term memory (LSTM), and comparisons with the current results. However, the most important component of future work lies in acquiring additional data from more subjects, especially with respect to activities such as jumping and running, in order to address class imbalance and enhance the generalization of models.

## 5. Conclusions

This paper explored the hypothesis of using sensor fusion-based features to improve the prediction of machine learning models for human activity recognition, especially regarding the detection of fall activities. Analyses of both time and frequency features derived from both accelerometer and barometric altimeter signal data in order to build comprehensive and interpretable models were conducted. Using $F_{1}$ score, 5-Fold validation, and ROC-AUC metrics, all four traditional machine learning methods (RF, SVM, XGB, and LR), as well as their combined predictions using majority voter, produced better results when using sensor fusion-based features based on all metrics. Cross-data generalization proved the robustness of RF and XGB models specifically. Although the results confirm that accelerometer signals individually hold more significance when it comes to fall detection, our studies suggest that combining barometric altimeter data improves the model’s overall performance and helps in gaining a deeper understanding of the motion characteristics of signals describing human movements.

## Figures and Tables

**Table 1:** Human activity recognition model performance using only accelerometer data.

		Falling	Jumping	Running	Sitting	Walking	Weighted	Macro	ADL	FALL	Weighted	Macro
RF	P	0.93	0.82	1.00	0.96	0.90	0.95	0.92	0.96	0.92	0.95	0.94
	R	0.93	0.69	0.80	0.97	0.90	0.95	0.86	0.97	0.92	0.95	0.94
	$F_{1}$	0.93	0.75	0.89	0.96	0.90	0.95 ± 0.005	0.89 ± 0.018	0.97	0.92	0.95 ± 0.008	0.94 ± 0.012
	5-F							0.96				0.96
	ROC							0.98				0.98
SVM	P	0.89	0.75	1.00	0.96	0.82	0.93	0.88	0.93	0.78	0.89	0.86
	R	0.93	0.69	0.70	0.95	0.90	0.93	0.83	0.90	0.85	0.88	0.87
	$F_{1}$	0.91	0.72	0.82	0.96	0.86	0.92 ± 0.012	0.85 ± 0.015	0.91	0.81	0.88 ± 0.011	0.86 ± 0.017
	5-F							0.94				0.94
	ROC							0.96				0.94
XGB	P	0.92	0.79	1.00	0.97	0.90	0.95	0.92	0.96	0.93	0.95	0.95
	R	0.93	0.85	0.70	0.97	0.90	0.95	0.87	0.97	0.92	0.95	0.94
	$F_{1}$	0.93	0.81	0.82	0.97	0.90	0.95 ± 0.004	0.89 ± 0.019	0.97	0.92	0.95 ± 0.005	0.95 ± 0.008
	5-F							0.96				0.96
	ROC							0.99				0.98
LR	P	0.88	0.79	1.00	0.97	0.75	0.93	0.88	0.93	0.76	0.88	0.84
	R	0.93	0.85	0.70	0.94	0.90	0.93	0.86	0.88	0.84	0.87	0.86
	$F_{1}$	0.90	0.81	0.82	0.95	0.82	0.91 ± 0.016	0.84 ± 0.026	0.90	0.80	0.87 ± 0.012	0.85 ± 0.019
	5-F							0.93				0.93
	ROC							0.97				0.93
MV	P	0.92	0.92	1.00	0.96	0.82	0.95	0.92	0.95	0.93	0.94	0.94
	R	0.92	0.85	0.80	0.97	0.90	0.95	0.89	0.97	0.89	0.94	0.93
	$F_{1}$	0.92	0.88	0.89	0.96	0.86	0.95 ± 0.007	0.91 ± 0.010	0.96	0.91	0.94 ± 0.006	0.93 ± 0.009
	5-F							0.96				0.96
	ROC							0.97				0.97

**Table 2:** Human activity recognition model performance using only barometric altimeter data.

		Falling	Jumping	Running	Sitting	Walking	Weighted	Macro	ADL	FALL	Weighted	Macro
RF	P	0.87	0.12	0.23	0.86	0.57	0.82	0.46	0.90	0.87	0.89	0.88
	R	0.80	0.12	0.14	0.94	0.36	0.86	0.42	0.95	0.75	0.89	0.85
	$F_{1}$	0.83	0.12	0.17	0.90	0.44	0.86 ± 0.02	0.51 ± 0.027	0.92	0.81	0.89 ± 0.015	0.86 ± 0.024
	5-F							0.96				0.96
	ROC							0.94				0.96
SVM	P	0.68	0.14	0.21	0.93	0.39	0.82	0.47	0.93	0.69	0.85	0.81
	R	0.87	0.67	0.53	0.67	0.64	0.72	0.67	0.83	0.85	0.84	0.84
	$F_{1}$	0.77	0.23	0.30	0.78	0.48	0.75 ± 0.016	0.51 ± 0.010	0.88	0.76	0.84 ± 0.025	0.82 ± 0.033
	5-F							0.94				0.94
	ROC							0.93				0.92
XGB	P	0.85	0.17	1.00	0.89	0.50	0.86	0.68	0.91	0.82	0.89	0.87
	R	0.84	0.11	0.13	0.93	0.55	0.87	0.51	0.92	0.80	0.89	0.86
	$F_{1}$	0.85	0.13	0.24	0.91	0.52	0.86 ± 0.022	0.55 ± 0.060	0.92	0.81	0.90 ± 0.018	0.87 ± 0.027
	5-F							0.96				0.96
	ROC							0.95				0.95
LR	P	0.69	0.12	0.20	0.91	0.43	0.81	0.47	0.93	0.70	0.86	0.81
	R	0.81	0.56	0.60	0.68	0.82	0.71	0.69	0.84	0.85	0.84	0.85
	$F_{1}$	0.75	0.20	0.30	0.78	0.56	0.74 ± 0.027	0.52 ± 0.024	0.88	0.77	0.84 ± 0.029	0.82 ± 0.037
	5-F							0.93				0.93
	ROC							0.89				0.92
MV	P	0.87	0.40	0.50	0.88	0.50	0.85	0.63	0.92	0.82	0.89	0.87
	R	0.82	0.22	0.07	0.93	0.64	0.86	0.53	0.92	0.82	0.89	0.87
	$F_{1}$	0.84	0.29	0.12	0.91	0.56	0.86 ± 0.020	0.58 ± 0.047	0.92	0.82	0.89 ± 0.019	0.87 ± 0.028
	5-F							0.96				0.96
	ROC							0.94				0.95

**Table 3:** Human activity recognition model performance using sensor fusion of accelerometer and barometric altimeter data.

		Falling	Jumping	Running	Sitting	Walking	Weighted	Macro	ADL	FALL	Weighted	Macro
RF	P	0.91	1.00	0.92	0.98	1.00	0.96	0.96	0.97	0.96	0.97	0.96
	R	0.97	0.91	1.00	0.96	0.89	0.96	0.94	0.98	0.94	0.97	0.96
	$F_{1}$	0.94	0.95	0.96	0.97	0.94	0.96 ± 0.010	0.93 ± 0.023	0.98	0.95	0.97 ± 0.010	0.96 ± 0.013
	5-F							0.96				0.96
	ROC							0.98				0.98
SVM	P	0.87	0.90	0.85	0.97	0.80	0.93	0.88	0.96	0.84	0.93	0.90
	R	0.93	0.82	0.92	0.93	0.89	0.93	0.90	0.93	0.92	0.92	0.92
	$F_{1}$	0.90	0.86	0.88	0.95	0.84	0.94 ± 0.010	0.89 ± 0.026	0.94	0.88	0.93 ± 0.010	0.91 ± 0.014
	5-F							0.94				0.93
	ROC							0.96				0.96
XGB	P	0.90	0.91	0.92	0.98	1.00	0.95	0.94	0.97	0.94	0.97	0.96
	R	0.95	0.91	1.00	0.95	0.89	0.95	0.94	0.98	0.94	0.97	0.96
	$F_{1}$	0.93	0.91	0.96	0.97	0.94	0.96 ± 0.008	0.93 ± 0.034	0.98	0.94	0.97 ± 0.009	0.96 ± 0.012
	5-F							0.97				0.96
	ROC							0.99				0.98
LR	P	0.87	1.00	0.86	0.96	0.73	0.93	0.88	0.96	0.82	0.92	0.89
	R	0.92	0.82	1.00	0.93	0.89	0.93	0.91	0.91	0.91	0.91	0.91
	$F_{1}$	0.90	0.90	0.92	0.95	0.80	0.93 ± 0.010	0.89 ± 0.016	0.94	0.86	0.92 ± 0.009	0.91 ± 0.014
	5-F							0.93				0.93
	ROC							0.99				0.95
MV	P	0.91	1.00	0.92	0.98	1.00	0.96	0.96	0.97	0.93	0.96	0.95
	R	0.95	0.91	1.00	0.96	0.89	0.96	0.94	0.97	0.93	0.96	0.95
	$F_{1}$	0.93	0.95	0.96	0.97	0.94	0.96 ± 0.009	0.94 ± 0.015	0.97	0.93	0.96 ± 0.010	0.95 ± 0.013
	5-F							0.96				0.96
	ROC							0.99				0.97

**Table 4:** Cross-dataset generalization results for different classifiers and sensor modalities. Values represent mean ± standard deviation across datasets.

Sensor	ML Method	Weighted $F_{1}$	Macro $F_{1}$	ROC-AUC
Acc	RF	0.824 ± 0.093	0.715 ± 0.104	0.891
	SVM	0.598 ± 0.302	0.527 ± 0.262	0.662
	XGB	0.812 ± 0.005	0.716 ± 0.015	0.852
	LR	0.613 ± 0.307	0.568 ± 0.270	0.680
	MV	0.653 ± 0.203	0.580 ± 0.270	0.844
Alt	RF	0.818 ± 0.090	0.769 ± 0.147	0.824
	SVM	0.786 ± 0.089	0.691 ± 0.102	0.813
	XGB	0.813 ± 0.078	0.771 ± 0.136	0.830
	LR	0.786 ± 0.098	0.692 ± 0.117	0.802
	MV	0.792 ± 0.136	0.743 ± 0.190	0.820
Acc + Alt	RF	0.864 ± 0.004	0.781 ± 0.033	0.927
	SVM	0.626 ± 0.326	0.592 ± 0.304	0.740
	XGB	0.885 ± 0.049	0.823 ± 0.044	0.930
	LR	0.634 ± 0.344	0.606 ± 0.328	0.733
	MV	0.733 ± 0.201	0.673 ± 0.206	0.934

**Table 5:** ANOVA and Tukey HSD results for classifier performance across sensor modalities. ANOVA *p*-values indicate overall significance; Tukey HSD lists pairs of classifiers with significant differences.

Classification Task	Sensor	ANOVA *p*-Value ($F_{1}$)	Tukey HSD (Significant Pairs)
Multiclass (falling, jumping, running, sitting, and walking)	Acc	0.0000	RF and SVM RF and LR SVM and XGB XGB and LR
	Alt	0.05096	None
	Acc + Alt	0.01431	None
Binary (ADL vs. FALL)	Acc	0.00000	RF and LR RF and SVM SVM and XGB XGB and LR
	Alt	0.02357	None
	Acc + Alt	0.00001	RF and LR RF and SVM SVM and XGB XGB and LR

## Data Availability

The raw data supporting the conclusions of this article will be made available by the authors on request.
